# (3a*RS*,4*SR*,7*RS*,7a*SR*)-2-(Tricyclo­[3.3.1.1^3,7^]decan-1-yl)-4,5,6,7-tetra­hydro-4,7-epoxy­isoindoline-1,3-dione

**DOI:** 10.1107/S1600536810016971

**Published:** 2010-05-15

**Authors:** Zaiyou Tan, Lin Luo, Erjia Zhu, Ruisi Yan, Zhuohui Lin

**Affiliations:** a Department of Physical Chemistry, Guangdong Pharmaceutical University, Guangzhou, Guangdong 510006, People’s Republic of China

## Abstract

The title compound, C_18_H_23_NO_3_, the adamantane derivative of norcantharidin, which is itself derived from cantharidin, crystallized with three independent mol­ecules in the asymmetric unit. In the crystal, mol­ecules are linked by inter­molecular C—H⋯O inter­actions, leading to the formation of a supra­molecular two-dimensional network.

## Related literature

For the synthesis and anti­cancer activity of norcantharimides, see: Hill *et al.* (2007 ▶); Tan (2009 ▶). For the synthesis and anti­cancer activity of norcantharidin, see: Shimi & Zaki (1982 ▶). For background to the medicinal uses of catharidin, see: Wang (1989 ▶). For the crystal structure of the phenyl derivative of norcantharidin, see: Zhu & Lin (2009 ▶).
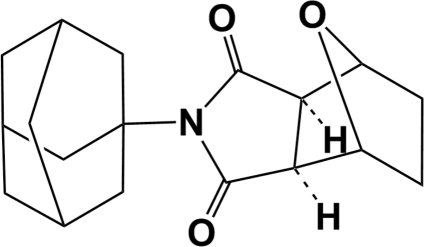


## Experimental

### 

#### Crystal data

C_18_H_23_NO_3_
*M* *_r_* = 301.37
Triclinic, 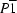

*a* = 12.2216 (4) Å
*b* = 12.3465 (4) Å
*c* = 16.1646 (6) Å
α = 77.057 (3)°
β = 89.906 (3)°
γ = 69.190 (3)°
*V* = 2213.95 (14) Å^3^
*Z* = 6
Cu *K*α radiation
μ = 0.74 mm^−1^
*T* = 100 K
0.2 × 0.2 × 0.1 mm

#### Data collection

Oxford Diffraction Xcalibur Onyx Nova diffractometer
Absorption correction: multi-scan (*CrysAlis PRO*; Oxford Diffraction, 2009 ▶) *T*_min_ = 0.891, *T*_max_ = 1.0
15170 measured reflections
7996 independent reflections
7431 reflections with *I* > 2σ(*I*)
*R*_int_ = 0.021

#### Refinement

*R*[*F* ^2^ > 2σ(*F* ^2^)] = 0.038
*wR*(*F* ^2^) = 0.098
*S* = 1.02
7996 reflections
595 parameters
H-atom parameters constrained
Δρ_max_ = 0.31 e Å^−3^
Δρ_min_ = −0.22 e Å^−3^

### 

Data collection: *CrysAlis PRO* (Oxford Diffraction, 2009 ▶); cell refinement: *CrysAlis PRO*; data reduction: *CrysAlis PRO*; program(s) used to solve structure: *SHELXS97* (Sheldrick, 2008 ▶); program(s) used to refine structure: *SHELXL97* (Sheldrick, 2008 ▶); molecular graphics: *OLEX2* (Dolomanov *et al.*, 2009 ▶); software used to prepare material for publication: *OLEX2*.

## Supplementary Material

Crystal structure: contains datablocks I, global. DOI: 10.1107/S1600536810016971/su2162sup1.cif

Structure factors: contains datablocks I. DOI: 10.1107/S1600536810016971/su2162Isup2.hkl

Additional supplementary materials:  crystallographic information; 3D view; checkCIF report

## Figures and Tables

**Table 1:** Hydrogen-bond geometry (Å, °)

*D*—H⋯*A*	*D*—H	H⋯*A*	*D*⋯*A*	*D*—H⋯*A*
C2—H2⋯O2^i^	0.98	2.53	3.3332 (17)	140
C5—H5*B*⋯O5^ii^	0.97	2.59	3.4463 (18)	148
C37—H37⋯O5^iii^	0.98	2.38	3.3239 (16)	161
C41—H41*B*⋯O3^iv^	0.97	2.51	3.4788 (18)	176

Symmetry codes: (i) 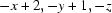; (ii) 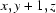; (iii) 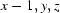; (iv) 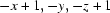.

**Fig. 1.**
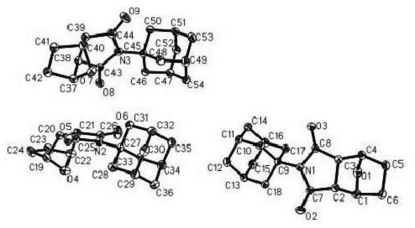
A view of the molecular structure of the three independent molecules of compound (III). The displacement ellipsoids are drawn at the 50% probability level.

**Fig. 2.**
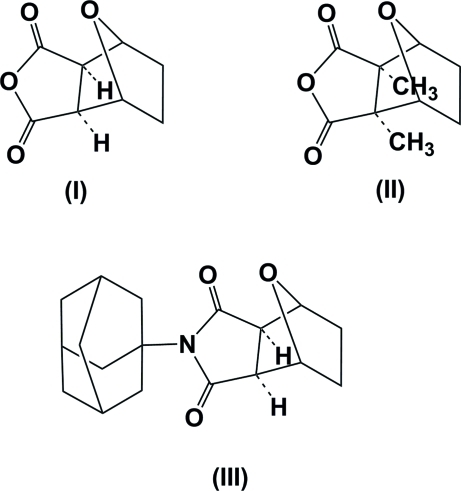
Schematic views of (I), (II) and (III).

## supplementary crystallographic information

### Comment

Norcantharidine {(I) = 7-oxabicyclo[2.2.1]heptane-2,3-dicarboxylic anhydride}, derived from cantharidin {(II) = 2,6-Dimethyl-4,10-dioxatricyclo-[5.2.1.02,6]decane-3,5-dione}, is a low toxicity anticancer drug (Shimi & Zaki, 1982). A number of norcantharimides have been synthesized from norcantharidin and have been shown to possess interesting anticancer activity (Hill *et al.*, 2007; Tan, 2009). In order to study the relationship between the anticancer activity of norcantharidin and the adamantane norcantharimide derivative, the title compound (III) was synthesized and its crystal structure is reported on here.

Compound (III) crystallized with three independent molecules per asymmetric unit, Fig. 1. The bond distances and angles in the three independent molecules are very similar and close to those observed in a similar compound, the phenyl derivative of norcantharidin (Zhu & Lin, 2009).

In the crystal structure the individual molecules are linked via C-H···O interactions leading to the formation of a supramolecular network (Table 1).

### Experimental

Norcantharidin (1.0 g) and adamantine (0.9 g) were dissolved in DMF (10 mL) and the mixture was heated to reflux with stirring for 18 h. The solvent was then evaporated off and the crude product remaining was dissolved in warm acetone (10 mL) and cooled rapidly. The clear solution obtained was left undisturbed at 255 K for several days and gave finally colourless crystals of the title compound.

### Refinement

The H-atoms were included in calculated positions and treated as riding atoms: C-H = 0.97 - 0.98 Å, with U_iso_(H) = 1.2U_eq_(parent C-atom).

### Crystal data

**Table:**

C_18_H_23_NO_3_	*Z* = 6
*M**_r_* = 301.37	*F*(000) = 972
Triclinic, *P*1	*D*_x_ = 1.356 Mg m^−^^3^
Hall symbol: -P 1	Cu *K*α radiation, λ = 1.5418 Å
*a* = 12.2216 (4) Å	Cell parameters from 12892 reflections
*b* = 12.3465 (4) Å	θ = 2.8–71.2°
*c* = 16.1646 (6) Å	µ = 0.74 mm^−^^1^
α = 77.057 (3)°	*T* = 100 K
β = 89.906 (3)°	Plate, colourless
γ = 69.190 (3)°	0.2 × 0.2 × 0.1 mm
*V* = 2213.95 (14) Å^3^	

### Data collection

**Table:**

Oxford Diffraction Xcalibur Onyx Nova diffractometer	7996 independent reflections
Radiation source: fine-focus sealed tube	7431 reflections with *I* > 2σ(*I*)
mirror	*R*_int_ = 0.021
Detector resolution: 8.2417 pixels mm^-1^	θ_max_ = 68.3°, θ_min_ = 2.8°
ω scans	*h* = −12→14
Absorption correction: multi-scan (*CrysAlis PRO*; Oxford Diffraction, 2009)	*k* = −14→13
*T*_min_ = 0.891, *T*_max_ = 1.0	*l* = −19→15
15170 measured reflections	

### Refinement

**Table:**

Refinement on *F*^2^	Primary atom site location: structure-invariant direct methods
Least-squares matrix: full	Secondary atom site location: difference Fourier map
*R*[*F*^2^ > 2σ(*F*^2^)] = 0.038	Hydrogen site location: inferred from neighbouring sites
*wR*(*F*^2^) = 0.098	H-atom parameters constrained
*S* = 1.02	*w* = 1/[σ^2^(*F*_o_^2^) + (0.0465*P*)^2^ + 1.1406*P*] where *P* = (*F*_o_^2^ + 2*F*_c_^2^)/3
7996 reflections	(Δ/σ)_max_ < 0.001
595 parameters	Δρ_max_ = 0.31 e Å^−^^3^
0 restraints	Δρ_min_ = −0.22 e Å^−^^3^

### Special details

**Table:**

Experimental. CrysAlisPro (Oxford Diffraction, 2009) Empirical absorption correction using spherical harmonics, implemented in SCALE3 ABSPACK scaling algorithm.
Geometry. All esds (except the esd in the dihedral angle between two l.s. planes) are estimated using the full covariance matrix. The cell esds are taken into account individually in the estimation of esds in distances, angles and torsion angles; correlations between esds in cell parameters are only used when they are defined by crystal symmetry. An approximate (isotropic) treatment of cell esds is used for estimating esds involving l.s. planes.
Refinement. Refinement of *F*^2^ against ALL reflections. The weighted *R*-factor wR and goodness of fit *S* are based on *F*^2^, conventional *R*-factors *R* are based on *F*, with *F* set to zero for negative *F*^2^. The threshold expression of *F*^2^ > σ(*F*^2^) is used only for calculating *R*-factors(gt) etc. and is not relevant to the choice of reflections for refinement. *R*-factors based on *F*^2^ are statistically about twice as large as those based on *F*, and *R*- factors based on ALL data will be even larger.

### Fractional atomic coordinates and isotropic or equivalent isotropic displacement parameters (Å^2^)

**Table:**

	*x*	*y*	*z*	*U*_iso_*/*U*_eq_	
N1	0.80521 (9)	0.52188 (9)	0.17059 (6)	0.0172 (2)	
N2	1.12676 (9)	0.07469 (9)	0.18785 (7)	0.0200 (2)	
N3	0.19346 (10)	0.00814 (10)	0.51576 (7)	0.0213 (2)	
O1	0.94100 (8)	0.70652 (8)	0.16579 (6)	0.0255 (2)	
O2	0.82947 (8)	0.54694 (10)	0.02538 (6)	0.0284 (2)	
O3	0.84965 (8)	0.48301 (9)	0.31659 (6)	0.0235 (2)	
O4	0.91864 (9)	0.10809 (9)	0.05730 (6)	0.0281 (2)	
O5	1.18443 (8)	−0.12995 (8)	0.20189 (6)	0.0263 (2)	
O6	1.01337 (9)	0.26134 (9)	0.20870 (8)	0.0375 (3)	
O7	0.34150 (8)	−0.22687 (9)	0.44669 (6)	0.0269 (2)	
O8	0.06551 (9)	0.05988 (8)	0.39398 (6)	0.0279 (2)	
O9	0.31865 (11)	−0.10781 (9)	0.63688 (7)	0.0394 (3)	
C1	0.99762 (12)	0.65254 (12)	0.09882 (9)	0.0236 (3)	
H1	0.9631	0.6989	0.0413	0.028*	
C2	0.98732 (11)	0.52836 (11)	0.12273 (8)	0.0194 (3)	
H2	1.0496	0.4684	0.1014	0.023*	
C3	0.99635 (11)	0.50262 (11)	0.22009 (8)	0.0182 (3)	
H3	1.0611	0.4284	0.2466	0.022*	
C4	1.01471 (11)	0.61378 (11)	0.23561 (8)	0.0215 (3)	
H4	0.9952	0.6284	0.2919	0.026*	
C5	1.13899 (12)	0.60702 (12)	0.21473 (9)	0.0240 (3)	
H5A	1.1967	0.5278	0.2380	0.029*	
H5B	1.1606	0.6649	0.2358	0.029*	
C6	1.12610 (12)	0.63743 (13)	0.11592 (9)	0.0257 (3)	
H6A	1.1792	0.5730	0.0938	0.031*	
H6B	1.1396	0.7104	0.0917	0.031*	
C7	0.86623 (11)	0.53358 (11)	0.09782 (8)	0.0194 (3)	
C8	0.87785 (11)	0.49970 (11)	0.24416 (8)	0.0182 (3)	
C9	0.67839 (11)	0.53414 (11)	0.17438 (8)	0.0173 (3)	
C10	0.67026 (11)	0.41931 (11)	0.23060 (8)	0.0191 (3)	
H10A	0.7095	0.3525	0.2056	0.023*	
H10B	0.7092	0.4029	0.2867	0.023*	
C11	0.54068 (12)	0.43378 (12)	0.23847 (8)	0.0217 (3)	
H11	0.5360	0.3609	0.2750	0.026*	
C12	0.47996 (12)	0.45685 (12)	0.15020 (9)	0.0225 (3)	
H12A	0.3983	0.4656	0.1550	0.027*	
H12B	0.5178	0.3899	0.1250	0.027*	
C13	0.48759 (11)	0.57089 (11)	0.09362 (8)	0.0207 (3)	
H13	0.4494	0.5853	0.0369	0.025*	
C14	0.47916 (12)	0.53976 (12)	0.27779 (9)	0.0234 (3)	
H14A	0.3977	0.5484	0.2839	0.028*	
H14B	0.5170	0.5255	0.3340	0.028*	
C15	0.42546 (11)	0.67717 (12)	0.13211 (9)	0.0233 (3)	
H15A	0.3434	0.6873	0.1364	0.028*	
H15B	0.4294	0.7495	0.0958	0.028*	
C16	0.48527 (11)	0.65468 (12)	0.22058 (9)	0.0225 (3)	
H16	0.4454	0.7222	0.2458	0.027*	
C17	0.61458 (11)	0.64107 (11)	0.21266 (8)	0.0201 (3)	
H17A	0.6190	0.7133	0.1764	0.024*	
H17B	0.6525	0.6289	0.2684	0.024*	
C18	0.61753 (11)	0.55623 (11)	0.08571 (8)	0.0186 (3)	
H18A	0.6567	0.4895	0.0606	0.022*	
H18B	0.6225	0.6277	0.0488	0.022*	
C19	0.92595 (12)	−0.00744 (13)	0.10737 (9)	0.0252 (3)	
H19	0.9715	−0.0743	0.0833	0.030*	
C20	0.97899 (11)	−0.00961 (11)	0.19448 (8)	0.0210 (3)	
H20	0.9604	−0.0637	0.2417	0.025*	
C21	0.92201 (11)	0.12145 (11)	0.19812 (8)	0.0214 (3)	
H21	0.8752	0.1322	0.2470	0.026*	
C22	0.84732 (12)	0.17727 (13)	0.11202 (9)	0.0263 (3)	
H22	0.8282	0.2634	0.0920	0.032*	
C23	0.73999 (13)	0.14035 (14)	0.11709 (10)	0.0332 (3)	
H23A	0.6828	0.1855	0.0687	0.040*	
H23B	0.7027	0.1500	0.1694	0.040*	
C24	0.79682 (13)	0.00667 (15)	0.11539 (10)	0.0333 (3)	
H24A	0.7643	−0.0097	0.0670	0.040*	
H24B	0.7872	−0.0450	0.1675	0.040*	
C25	1.10853 (12)	−0.03268 (11)	0.19481 (8)	0.0205 (3)	
C26	1.02306 (12)	0.16407 (12)	0.19936 (9)	0.0239 (3)	
C27	1.24486 (11)	0.08634 (11)	0.17863 (8)	0.0191 (3)	
C28	1.31391 (13)	0.00955 (13)	0.11948 (9)	0.0268 (3)	
H28A	1.2709	0.0363	0.0638	0.032*	
H28B	1.3229	−0.0730	0.1425	0.032*	
C29	1.43579 (13)	0.01918 (13)	0.11085 (9)	0.0284 (3)	
H29	1.4799	−0.0320	0.0744	0.034*	
C30	1.50192 (12)	−0.02155 (12)	0.19902 (10)	0.0276 (3)	
H30A	1.5115	−0.1039	0.2239	0.033*	
H30B	1.5794	−0.0171	0.1940	0.033*	
C31	1.31319 (11)	0.04571 (12)	0.26620 (8)	0.0215 (3)	
H31A	1.3234	−0.0366	0.2915	0.026*	
H31B	1.2696	0.0944	0.3035	0.026*	
C32	1.43417 (12)	0.05747 (12)	0.25655 (9)	0.0240 (3)	
H32	1.4779	0.0319	0.3126	0.029*	
C33	1.23082 (12)	0.21661 (12)	0.13862 (9)	0.0252 (3)	
H33A	1.1865	0.2433	0.0834	0.030*	
H33B	1.1876	0.2674	0.1747	0.030*	
C34	1.35213 (12)	0.22691 (12)	0.12811 (9)	0.0250 (3)	
H34	1.3420	0.3102	0.1028	0.030*	
C35	1.41880 (12)	0.18701 (12)	0.21611 (9)	0.0247 (3)	
H35A	1.3756	0.2380	0.2522	0.030*	
H35B	1.4952	0.1941	0.2109	0.030*	
C36	1.42049 (14)	0.14850 (13)	0.07055 (9)	0.0291 (3)	
H36A	1.3781	0.1740	0.0147	0.035*	
H36B	1.4968	0.1555	0.0639	0.035*	
C37	0.22597 (12)	−0.21048 (12)	0.41329 (8)	0.0235 (3)	
H37	0.2070	−0.1680	0.3531	0.028*	
C38	0.14640 (12)	−0.14428 (12)	0.47431 (8)	0.0215 (3)	
H38	0.0721	−0.1578	0.4780	0.026*	
C39	0.22433 (13)	−0.19952 (12)	0.55816 (8)	0.0240 (3)	
H39	0.1871	−0.2365	0.6041	0.029*	
C40	0.33389 (12)	−0.29000 (12)	0.53188 (9)	0.0248 (3)	
H40	0.4047	−0.3133	0.5702	0.030*	
C41	0.30524 (13)	−0.39584 (12)	0.51712 (9)	0.0261 (3)	
H41A	0.3757	−0.4614	0.5116	0.031*	
H41B	0.2630	−0.4240	0.5626	0.031*	
C42	0.22677 (13)	−0.33821 (12)	0.43193 (9)	0.0265 (3)	
H42A	0.2609	−0.3778	0.3875	0.032*	
H42B	0.1483	−0.3394	0.4384	0.032*	
C43	0.12808 (11)	−0.01332 (12)	0.45425 (8)	0.0207 (3)	
C44	0.25253 (13)	−0.09775 (12)	0.57748 (9)	0.0265 (3)	
C45	0.21348 (11)	0.12225 (11)	0.51530 (8)	0.0203 (3)	
C46	0.13275 (12)	0.22885 (11)	0.44621 (8)	0.0216 (3)	
H46A	0.1461	0.2118	0.3905	0.026*	
H46B	0.0512	0.2421	0.4560	0.026*	
C47	0.15888 (12)	0.34125 (12)	0.44895 (8)	0.0238 (3)	
H47	0.1072	0.4087	0.4050	0.029*	
C48	0.34207 (12)	0.10238 (12)	0.49780 (9)	0.0240 (3)	
H48A	0.3564	0.0841	0.4425	0.029*	
H48B	0.3940	0.0352	0.5407	0.029*	
C49	0.36785 (12)	0.21503 (13)	0.49950 (9)	0.0268 (3)	
H49	0.4500	0.2018	0.4886	0.032*	
C50	0.18965 (12)	0.15242 (12)	0.60276 (8)	0.0232 (3)	
H50A	0.1080	0.1668	0.6129	0.028*	
H50B	0.2383	0.0857	0.6474	0.028*	
C51	0.21730 (12)	0.26423 (12)	0.60447 (8)	0.0232 (3)	
H51	0.2039	0.2820	0.6606	0.028*	
C52	0.13667 (12)	0.36989 (12)	0.53621 (9)	0.0243 (3)	
H52A	0.0552	0.3843	0.5470	0.029*	
H52B	0.1525	0.4412	0.5374	0.029*	
C53	0.34583 (13)	0.24245 (12)	0.58718 (9)	0.0268 (3)	
H53A	0.3971	0.1759	0.6308	0.032*	
H53B	0.3633	0.3126	0.5891	0.032*	
C54	0.28674 (13)	0.32085 (13)	0.43116 (9)	0.0278 (3)	
H54A	0.3003	0.3045	0.3753	0.033*	
H54B	0.3033	0.3919	0.4318	0.033*	

### Atomic displacement parameters (Å^2^)

**Table:**

	*U*^11^	*U*^22^	*U*^33^	*U*^12^	*U*^13^	*U*^23^
O1	0.0230 (5)	0.0189 (5)	0.0326 (5)	−0.0060 (4)	0.0003 (4)	−0.0050 (4)
O2	0.0247 (5)	0.0459 (6)	0.0176 (5)	−0.0156 (4)	0.0031 (4)	−0.0095 (4)
O3	0.0252 (5)	0.0321 (5)	0.0165 (5)	−0.0139 (4)	0.0027 (4)	−0.0064 (4)
O4	0.0275 (5)	0.0336 (5)	0.0204 (5)	−0.0078 (4)	0.0028 (4)	−0.0065 (4)
O5	0.0238 (5)	0.0176 (5)	0.0350 (5)	−0.0065 (4)	0.0019 (4)	−0.0034 (4)
O6	0.0328 (6)	0.0246 (5)	0.0626 (8)	−0.0128 (4)	0.0129 (5)	−0.0214 (5)
O7	0.0256 (5)	0.0265 (5)	0.0292 (5)	−0.0122 (4)	0.0054 (4)	−0.0034 (4)
O8	0.0305 (5)	0.0235 (5)	0.0278 (5)	−0.0092 (4)	−0.0072 (4)	−0.0035 (4)
O9	0.0607 (8)	0.0221 (5)	0.0311 (6)	−0.0123 (5)	−0.0203 (5)	−0.0019 (4)
N1	0.0170 (5)	0.0194 (5)	0.0165 (5)	−0.0076 (4)	0.0019 (4)	−0.0054 (4)
N2	0.0201 (5)	0.0177 (5)	0.0227 (5)	−0.0074 (4)	−0.0001 (4)	−0.0051 (4)
N3	0.0269 (6)	0.0185 (5)	0.0187 (5)	−0.0090 (5)	−0.0011 (4)	−0.0036 (4)
C1	0.0230 (7)	0.0252 (7)	0.0220 (7)	−0.0107 (5)	0.0004 (5)	−0.0010 (5)
C2	0.0184 (6)	0.0215 (6)	0.0188 (6)	−0.0074 (5)	0.0027 (5)	−0.0057 (5)
C3	0.0185 (6)	0.0182 (6)	0.0183 (6)	−0.0067 (5)	0.0009 (5)	−0.0050 (5)
C4	0.0233 (7)	0.0213 (6)	0.0218 (6)	−0.0088 (5)	0.0017 (5)	−0.0077 (5)
C5	0.0236 (7)	0.0250 (7)	0.0271 (7)	−0.0128 (6)	0.0005 (5)	−0.0068 (5)
C6	0.0242 (7)	0.0296 (7)	0.0261 (7)	−0.0144 (6)	0.0024 (5)	−0.0043 (6)
C7	0.0216 (6)	0.0194 (6)	0.0178 (6)	−0.0073 (5)	0.0032 (5)	−0.0058 (5)
C8	0.0211 (6)	0.0162 (6)	0.0183 (6)	−0.0072 (5)	0.0009 (5)	−0.0053 (5)
C9	0.0166 (6)	0.0182 (6)	0.0184 (6)	−0.0074 (5)	0.0020 (5)	−0.0053 (5)
C10	0.0198 (6)	0.0186 (6)	0.0194 (6)	−0.0076 (5)	0.0020 (5)	−0.0043 (5)
C11	0.0229 (7)	0.0210 (6)	0.0231 (7)	−0.0113 (5)	0.0021 (5)	−0.0032 (5)
C12	0.0207 (6)	0.0235 (7)	0.0271 (7)	−0.0106 (5)	0.0022 (5)	−0.0092 (5)
C13	0.0188 (6)	0.0224 (6)	0.0212 (6)	−0.0074 (5)	−0.0010 (5)	−0.0059 (5)
C14	0.0199 (6)	0.0308 (7)	0.0221 (6)	−0.0111 (6)	0.0049 (5)	−0.0083 (6)
C15	0.0179 (6)	0.0217 (7)	0.0285 (7)	−0.0050 (5)	0.0014 (5)	−0.0064 (5)
C16	0.0199 (6)	0.0222 (7)	0.0273 (7)	−0.0064 (5)	0.0045 (5)	−0.0117 (5)
C17	0.0210 (6)	0.0199 (6)	0.0221 (6)	−0.0086 (5)	0.0034 (5)	−0.0084 (5)
C18	0.0190 (6)	0.0191 (6)	0.0183 (6)	−0.0071 (5)	0.0018 (5)	−0.0052 (5)
C19	0.0243 (7)	0.0288 (7)	0.0263 (7)	−0.0106 (6)	0.0033 (5)	−0.0127 (6)
C20	0.0228 (7)	0.0205 (6)	0.0208 (6)	−0.0096 (5)	0.0024 (5)	−0.0045 (5)
C21	0.0212 (6)	0.0219 (7)	0.0219 (6)	−0.0073 (5)	0.0043 (5)	−0.0077 (5)
C22	0.0223 (7)	0.0261 (7)	0.0258 (7)	−0.0031 (6)	0.0008 (5)	−0.0062 (6)
C23	0.0215 (7)	0.0418 (9)	0.0353 (8)	−0.0078 (6)	−0.0009 (6)	−0.0134 (7)
C24	0.0255 (7)	0.0443 (9)	0.0377 (8)	−0.0157 (7)	0.0014 (6)	−0.0201 (7)
C25	0.0242 (7)	0.0199 (7)	0.0184 (6)	−0.0100 (6)	0.0010 (5)	−0.0032 (5)
C26	0.0257 (7)	0.0217 (7)	0.0253 (7)	−0.0089 (5)	0.0027 (5)	−0.0070 (5)
C27	0.0202 (6)	0.0191 (6)	0.0199 (6)	−0.0094 (5)	0.0017 (5)	−0.0053 (5)
C28	0.0330 (8)	0.0294 (7)	0.0259 (7)	−0.0169 (6)	0.0089 (6)	−0.0132 (6)
C29	0.0342 (8)	0.0284 (7)	0.0298 (7)	−0.0152 (6)	0.0141 (6)	−0.0151 (6)
C30	0.0229 (7)	0.0216 (7)	0.0364 (8)	−0.0069 (6)	0.0073 (6)	−0.0053 (6)
C31	0.0221 (7)	0.0216 (6)	0.0198 (6)	−0.0074 (5)	0.0018 (5)	−0.0038 (5)
C32	0.0217 (7)	0.0262 (7)	0.0220 (6)	−0.0078 (5)	−0.0013 (5)	−0.0035 (5)
C33	0.0252 (7)	0.0206 (7)	0.0280 (7)	−0.0093 (5)	−0.0024 (6)	−0.0008 (5)
C34	0.0291 (7)	0.0194 (6)	0.0271 (7)	−0.0118 (6)	0.0007 (6)	−0.0016 (5)
C35	0.0240 (7)	0.0266 (7)	0.0281 (7)	−0.0124 (6)	0.0040 (5)	−0.0103 (6)
C36	0.0352 (8)	0.0346 (8)	0.0227 (7)	−0.0192 (7)	0.0071 (6)	−0.0063 (6)
C37	0.0276 (7)	0.0249 (7)	0.0189 (6)	−0.0106 (6)	0.0023 (5)	−0.0052 (5)
C38	0.0242 (7)	0.0223 (7)	0.0212 (6)	−0.0119 (5)	0.0029 (5)	−0.0060 (5)
C39	0.0346 (8)	0.0201 (6)	0.0185 (6)	−0.0124 (6)	0.0019 (5)	−0.0032 (5)
C40	0.0277 (7)	0.0214 (7)	0.0243 (7)	−0.0097 (6)	−0.0019 (5)	−0.0019 (5)
C41	0.0333 (7)	0.0212 (7)	0.0253 (7)	−0.0117 (6)	0.0031 (6)	−0.0060 (5)
C42	0.0322 (7)	0.0248 (7)	0.0253 (7)	−0.0118 (6)	0.0030 (6)	−0.0093 (6)
C43	0.0216 (6)	0.0231 (7)	0.0192 (6)	−0.0096 (5)	0.0030 (5)	−0.0062 (5)
C44	0.0372 (8)	0.0210 (7)	0.0205 (7)	−0.0104 (6)	−0.0028 (6)	−0.0035 (5)
C45	0.0244 (7)	0.0178 (6)	0.0199 (6)	−0.0086 (5)	0.0006 (5)	−0.0054 (5)
C46	0.0252 (7)	0.0208 (6)	0.0187 (6)	−0.0083 (5)	−0.0003 (5)	−0.0047 (5)
C47	0.0307 (7)	0.0178 (6)	0.0210 (7)	−0.0078 (5)	−0.0014 (5)	−0.0028 (5)
C48	0.0237 (7)	0.0229 (7)	0.0261 (7)	−0.0068 (5)	0.0013 (5)	−0.0100 (5)
C49	0.0228 (7)	0.0285 (7)	0.0344 (8)	−0.0119 (6)	0.0059 (6)	−0.0133 (6)
C50	0.0294 (7)	0.0219 (7)	0.0187 (6)	−0.0098 (6)	0.0021 (5)	−0.0045 (5)
C51	0.0310 (7)	0.0215 (7)	0.0175 (6)	−0.0085 (6)	0.0006 (5)	−0.0073 (5)
C52	0.0279 (7)	0.0194 (6)	0.0247 (7)	−0.0064 (5)	0.0012 (5)	−0.0072 (5)
C53	0.0300 (7)	0.0222 (7)	0.0294 (7)	−0.0099 (6)	−0.0043 (6)	−0.0077 (6)
C54	0.0389 (8)	0.0270 (7)	0.0243 (7)	−0.0187 (6)	0.0084 (6)	−0.0083 (6)

### Geometric parameters (Å, °)

**Table:**

O3—C8	1.2137 (16)	C49—H49	0.9800
O1—C4	1.4450 (16)	C43—C38	1.5091 (18)
O1—C1	1.4450 (17)	C50—C51	1.5393 (18)
O2—C7	1.2095 (16)	C50—H50A	0.9700
N1—C7	1.3993 (16)	C50—H50B	0.9700
N1—C8	1.4009 (16)	C47—C54	1.530 (2)
N1—C9	1.5053 (15)	C47—C52	1.5304 (19)
C8—C3	1.5098 (17)	C47—H47	0.9800
C18—C9	1.5383 (17)	C38—C39	1.5290 (18)
C18—C13	1.5416 (17)	C38—C37	1.5426 (18)
C18—H18A	0.9700	C38—H38	0.9800
C18—H18B	0.9700	C52—C51	1.5284 (18)
C9—C17	1.5385 (17)	C52—H52B	0.9700
C9—C10	1.5389 (17)	C52—H52A	0.9700
C10—C11	1.5378 (17)	C51—H51	0.9800
C10—H10A	0.9700	C54—H54B	0.9700
C10—H10B	0.9700	C54—H54A	0.9700
C11—C12	1.5292 (18)	C41—C40	1.5338 (18)
C11—C14	1.5342 (19)	C41—H41B	0.9700
C11—H11	0.9800	C41—H41A	0.9700
C7—C2	1.5096 (17)	C39—C44	1.5096 (18)
C1—C6	1.5319 (18)	C39—C40	1.539 (2)
C1—C2	1.5451 (18)	C39—H39	0.9800
C1—H1	0.9800	C37—H37	0.9800
C3—C2	1.5301 (17)	C40—H40	0.9800
C3—C4	1.5394 (17)	O4—C22	1.4417 (17)
C3—H3	0.9800	O4—C19	1.4457 (17)
C16—C15	1.5290 (18)	O5—C25	1.2096 (16)
C16—C14	1.5366 (19)	O6—C26	1.2079 (17)
C16—C17	1.5370 (17)	N2—C26	1.3986 (18)
C16—H16	0.9800	N2—C25	1.4009 (16)
C13—C15	1.5302 (18)	N2—C27	1.5044 (16)
C13—C12	1.5310 (18)	C31—C27	1.5300 (17)
C13—H13	0.9800	C31—C32	1.5405 (18)
C4—C5	1.5326 (18)	C31—H31A	0.9700
C4—H4	0.9800	C31—H31B	0.9700
C12—H12A	0.9700	C27—C28	1.5363 (18)
C12—H12B	0.9700	C27—C33	1.5405 (17)
C14—H14B	0.9700	C25—C20	1.5055 (18)
C14—H14A	0.9700	C32—C30	1.527 (2)
C5—C6	1.5514 (19)	C32—C35	1.5294 (19)
C5—H5A	0.9700	C32—H32	0.9800
C5—H5B	0.9700	C20—C21	1.5325 (18)
C17—H17B	0.9700	C20—C19	1.5419 (18)
C17—H17A	0.9700	C20—H20	0.9800
C6—H6B	0.9700	C33—C34	1.5385 (19)
C6—H6A	0.9700	C33—H33A	0.9700
C2—H2	0.9800	C33—H33B	0.9700
C15—H15B	0.9700	C35—C34	1.5279 (19)
C15—H15A	0.9700	C35—H35A	0.9700
O8—C43	1.2115 (16)	C35—H35B	0.9700
O7—C37	1.4411 (16)	C19—C24	1.5336 (19)
O7—C40	1.4431 (16)	C19—H19	0.9800
N3—C43	1.4031 (16)	C26—C21	1.5064 (18)
N3—C44	1.4047 (17)	C22—C23	1.531 (2)
N3—C45	1.5116 (16)	C22—C21	1.5403 (18)
O9—C44	1.2117 (17)	C22—H22	0.9800
C45—C48	1.5380 (18)	C36—C34	1.526 (2)
C45—C46	1.5413 (18)	C36—C29	1.527 (2)
C45—C50	1.5426 (18)	C36—H36B	0.9700
C46—C47	1.5403 (18)	C36—H36A	0.9700
C46—H46A	0.9700	C29—C30	1.527 (2)
C46—H46B	0.9700	C29—C28	1.5395 (19)
C53—C49	1.531 (2)	C29—H29	0.9800
C53—C51	1.533 (2)	C34—H34	0.9800
C53—H53B	0.9700	C30—H30B	0.9700
C53—H53A	0.9700	C30—H30A	0.9700
C48—C49	1.5372 (18)	C21—H21	0.9800
C48—H48B	0.9700	C28—H28A	0.9700
C48—H48A	0.9700	C28—H28B	0.9700
C42—C37	1.5343 (18)	C24—C23	1.553 (2)
C42—C41	1.5501 (19)	C24—H24B	0.9700
C42—H42A	0.9700	C24—H24A	0.9700
C42—H42B	0.9700	C23—H23A	0.9700
C49—C54	1.532 (2)	C23—H23B	0.9700
			
C4—O1—C1	96.08 (9)	C52—C47—H47	109.1
C7—N1—C8	110.70 (10)	C46—C47—H47	109.1
C7—N1—C9	127.44 (10)	C43—C38—C39	105.45 (10)
C8—N1—C9	121.83 (10)	C43—C38—C37	112.58 (11)
O3—C8—N1	125.44 (12)	C39—C38—C37	101.30 (11)
O3—C8—C3	124.66 (11)	C43—C38—H38	112.3
N1—C8—C3	109.88 (10)	C39—C38—H38	112.3
C9—C18—C13	109.69 (10)	C37—C38—H38	112.3
C9—C18—H18A	109.7	C51—C52—C47	108.81 (11)
C13—C18—H18A	109.7	C51—C52—H52B	109.9
C9—C18—H18B	109.7	C47—C52—H52B	109.9
C13—C18—H18B	109.7	C51—C52—H52A	109.9
H18A—C18—H18B	108.2	C47—C52—H52A	109.9
N1—C9—C18	112.19 (10)	H52B—C52—H52A	108.3
N1—C9—C17	107.99 (9)	C52—C51—C53	109.59 (11)
C18—C9—C17	108.63 (10)	C52—C51—C50	109.28 (11)
N1—C9—C10	109.67 (10)	C53—C51—C50	109.86 (11)
C18—C9—C10	108.23 (10)	C52—C51—H51	109.4
C17—C9—C10	110.13 (10)	C53—C51—H51	109.4
C11—C10—C9	109.88 (10)	C50—C51—H51	109.4
C11—C10—H10A	109.7	C47—C54—C49	109.20 (11)
C9—C10—H10A	109.7	C47—C54—H54B	109.8
C11—C10—H10B	109.7	C49—C54—H54B	109.8
C9—C10—H10B	109.7	C47—C54—H54A	109.8
H10A—C10—H10B	108.2	C49—C54—H54A	109.8
C12—C11—C14	109.26 (11)	H54B—C54—H54A	108.3
C12—C11—C10	109.68 (11)	C40—C41—C42	101.09 (10)
C14—C11—C10	109.36 (10)	C40—C41—H41B	111.6
C12—C11—H11	109.5	C42—C41—H41B	111.6
C14—C11—H11	109.5	C40—C41—H41A	111.6
C10—C11—H11	109.5	C42—C41—H41A	111.6
O2—C7—N1	126.42 (12)	H41B—C41—H41A	109.4
O2—C7—C2	124.02 (12)	C44—C39—C38	104.33 (10)
N1—C7—C2	109.56 (10)	C44—C39—C40	110.43 (12)
O1—C1—C6	102.94 (11)	C38—C39—C40	101.94 (11)
O1—C1—C2	102.52 (10)	C44—C39—H39	113.1
C6—C1—C2	108.78 (11)	C38—C39—H39	113.1
O1—C1—H1	113.8	C40—C39—H39	113.1
C6—C1—H1	113.8	O7—C37—C42	103.06 (10)
C2—C1—H1	113.8	O7—C37—C38	102.22 (10)
C8—C3—C2	104.59 (10)	C42—C37—C38	108.41 (11)
C8—C3—C4	111.95 (10)	O7—C37—H37	114.0
C2—C3—C4	101.72 (10)	C42—C37—H37	114.0
C8—C3—H3	112.6	C38—C37—H37	114.0
C2—C3—H3	112.6	O9—C44—N3	125.57 (12)
C4—C3—H3	112.6	O9—C44—C39	124.28 (12)
C15—C16—C14	109.64 (11)	N3—C44—C39	110.14 (11)
C15—C16—C17	109.31 (11)	O7—C40—C41	103.07 (11)
C14—C16—C17	109.33 (11)	O7—C40—C39	101.43 (10)
C15—C16—H16	109.5	C41—C40—C39	109.64 (11)
C14—C16—H16	109.5	O7—C40—H40	113.8
C17—C16—H16	109.5	C41—C40—H40	113.8
C15—C13—C12	109.96 (11)	C39—C40—H40	113.8
C15—C13—C18	109.69 (10)	C22—O4—C19	96.62 (10)
C12—C13—C18	109.63 (10)	C26—N2—C25	110.24 (11)
C15—C13—H13	109.2	C26—N2—C27	125.68 (10)
C12—C13—H13	109.2	C25—N2—C27	123.78 (10)
C18—C13—H13	109.2	C27—C31—C32	109.52 (10)
O1—C4—C5	103.21 (10)	C27—C31—H31A	109.8
O1—C4—C3	101.80 (10)	C32—C31—H31A	109.8
C5—C4—C3	108.98 (11)	C27—C31—H31B	109.8
O1—C4—H4	113.9	C32—C31—H31B	109.8
C5—C4—H4	113.9	H31A—C31—H31B	108.2
C3—C4—H4	113.9	N2—C27—C31	109.63 (10)
C11—C12—C13	109.15 (10)	N2—C27—C28	109.72 (10)
C11—C12—H12A	109.9	C31—C27—C28	109.83 (11)
C13—C12—H12A	109.9	N2—C27—C33	110.72 (10)
C11—C12—H12B	109.9	C31—C27—C33	109.24 (10)
C13—C12—H12B	109.9	C28—C27—C33	107.68 (11)
H12A—C12—H12B	108.3	O5—C25—N2	125.85 (12)
C11—C14—C16	109.85 (11)	O5—C25—C20	124.58 (12)
C11—C14—H14B	109.7	N2—C25—C20	109.56 (11)
C16—C14—H14B	109.7	C30—C32—C35	109.08 (11)
C11—C14—H14A	109.7	C30—C32—C31	108.84 (11)
C16—C14—H14A	109.7	C35—C32—C31	110.06 (11)
H14B—C14—H14A	108.2	C30—C32—H32	109.6
C4—C5—C6	101.48 (10)	C35—C32—H32	109.6
C4—C5—H5A	111.5	C31—C32—H32	109.6
C6—C5—H5A	111.5	C25—C20—C21	104.67 (10)
C4—C5—H5B	111.5	C25—C20—C19	112.13 (11)
C6—C5—H5B	111.5	C21—C20—C19	101.61 (10)
H5A—C5—H5B	109.3	C25—C20—H20	112.6
C16—C17—C9	109.91 (10)	C21—C20—H20	112.6
C16—C17—H17B	109.7	C19—C20—H20	112.6
C9—C17—H17B	109.7	C34—C33—C27	110.20 (11)
C16—C17—H17A	109.7	C34—C33—H33A	109.6
C9—C17—H17A	109.7	C27—C33—H33A	109.6
H17B—C17—H17A	108.2	C34—C33—H33B	109.6
C1—C6—C5	100.99 (11)	C27—C33—H33B	109.6
C1—C6—H6B	111.6	H33A—C33—H33B	108.1
C5—C6—H6B	111.6	C34—C35—C32	109.88 (11)
C1—C6—H6A	111.6	C34—C35—H35A	109.7
C5—C6—H6A	111.6	C32—C35—H35A	109.7
H6B—C6—H6A	109.4	C34—C35—H35B	109.7
C7—C2—C3	105.07 (10)	C32—C35—H35B	109.7
C7—C2—C1	112.12 (10)	H35A—C35—H35B	108.2
C3—C2—C1	101.40 (10)	O4—C19—C24	103.27 (11)
C7—C2—H2	112.5	O4—C19—C20	101.72 (10)
C3—C2—H2	112.5	C24—C19—C20	108.22 (11)
C1—C2—H2	112.5	O4—C19—H19	114.1
C16—C15—C13	109.13 (10)	C24—C19—H19	114.1
C16—C15—H15B	109.9	C20—C19—H19	114.1
C13—C15—H15B	109.9	O6—C26—N2	126.43 (13)
C16—C15—H15A	109.9	O6—C26—C21	123.98 (13)
C13—C15—H15A	109.9	N2—C26—C21	109.59 (11)
H15B—C15—H15A	108.3	O4—C22—C23	102.91 (11)
C37—O7—C40	96.41 (10)	O4—C22—C21	102.12 (10)
C43—N3—C44	110.48 (11)	C23—C22—C21	108.64 (12)
C43—N3—C45	127.74 (10)	O4—C22—H22	114.0
C44—N3—C45	121.44 (10)	C23—C22—H22	114.0
N3—C45—C48	107.97 (10)	C21—C22—H22	114.0
N3—C45—C46	112.37 (10)	C34—C36—C29	108.78 (11)
C48—C45—C46	108.73 (11)	C34—C36—H36B	109.9
N3—C45—C50	109.43 (10)	C29—C36—H36B	109.9
C48—C45—C50	110.04 (10)	C34—C36—H36A	109.9
C46—C45—C50	108.29 (11)	C29—C36—H36A	109.9
C47—C46—C45	109.43 (10)	H36B—C36—H36A	108.3
C47—C46—H46A	109.8	C36—C29—C30	110.30 (11)
C45—C46—H46A	109.8	C36—C29—C28	109.14 (12)
C47—C46—H46B	109.8	C30—C29—C28	109.12 (11)
C45—C46—H46B	109.8	C36—C29—H29	109.4
H46A—C46—H46B	108.2	C30—C29—H29	109.4
C49—C53—C51	109.88 (11)	C28—C29—H29	109.4
C49—C53—H53B	109.7	C36—C34—C35	110.01 (12)
C51—C53—H53B	109.7	C36—C34—C33	110.17 (11)
C49—C53—H53A	109.7	C35—C34—C33	108.44 (11)
C51—C53—H53A	109.7	C36—C34—H34	109.4
H53B—C53—H53A	108.2	C35—C34—H34	109.4
C49—C48—C45	109.92 (11)	C33—C34—H34	109.4
C49—C48—H48B	109.7	C29—C30—C32	110.06 (11)
C45—C48—H48B	109.7	C29—C30—H30B	109.6
C49—C48—H48A	109.7	C32—C30—H30B	109.6
C45—C48—H48A	109.7	C29—C30—H30A	109.6
H48B—C48—H48A	108.2	C32—C30—H30A	109.6
C37—C42—C41	101.46 (10)	H30B—C30—H30A	108.2
C37—C42—H42A	111.5	C26—C21—C20	104.72 (11)
C41—C42—H42A	111.5	C26—C21—C22	110.82 (11)
C37—C42—H42B	111.5	C20—C21—C22	101.73 (10)
C41—C42—H42B	111.5	C26—C21—H21	112.9
H42A—C42—H42B	109.3	C20—C21—H21	112.9
C53—C49—C54	109.20 (12)	C22—C21—H21	112.9
C53—C49—C48	109.20 (12)	C27—C28—C29	110.12 (11)
C54—C49—C48	109.71 (11)	C27—C28—H28A	109.6
C53—C49—H49	109.6	C29—C28—H28A	109.6
C54—C49—H49	109.6	C27—C28—H28B	109.6
C48—C49—H49	109.6	C29—C28—H28B	109.6
O8—C43—N3	126.65 (12)	H28A—C28—H28B	108.2
O8—C43—C38	123.90 (12)	C19—C24—C23	101.32 (12)
N3—C43—C38	109.45 (11)	C19—C24—H24B	111.5
C51—C50—C45	109.61 (11)	C23—C24—H24B	111.5
C51—C50—H50A	109.7	C19—C24—H24A	111.5
C45—C50—H50A	109.7	C23—C24—H24A	111.5
C51—C50—H50B	109.7	H24B—C24—H24A	109.3
C45—C50—H50B	109.7	C22—C23—C24	101.36 (11)
H50A—C50—H50B	108.2	C22—C23—H23A	111.5
C54—C47—C52	109.90 (11)	C24—C23—H23A	111.5
C54—C47—C46	109.51 (11)	C22—C23—H23B	111.5
C52—C47—C46	110.24 (11)	C24—C23—H23B	111.5
C54—C47—H47	109.1	H23A—C23—H23B	109.3

### Hydrogen-bond geometry (Å, °)

**Table:**

*D*—H···*A*	*D*—H	H···*A*	*D*···*A*	*D*—H···*A*
C2—H2···O2^i^	0.98	2.53	3.3332 (17)	140
C5—H5B···O5^ii^	0.97	2.59	3.4463 (18)	148
C37—H37···O5^iii^	0.98	2.38	3.3239 (16)	161
C41—H41B···O3^iv^	0.97	2.51	3.4788 (18)	176

Symmetry codes: (i) −*x*+2, −*y*+1, −*z*; (ii) *x*, *y*+1, *z*; (iii) *x*−1, *y*, *z*; (iv) −*x*+1, −*y*, −*z*+1.
